# Cyclist Orientation Estimation Using LiDAR Data

**DOI:** 10.3390/s23063096

**Published:** 2023-03-14

**Authors:** Hyoungwon Chang, Yanlei Gu, Igor Goncharenko, Li-Ta Hsu, Chinthaka Premachandra

**Affiliations:** 1 College of Information Science and Engineering, Ritsumeikan University, 1-1-1, Noji-higashi, Kusatsu 525-8577, Shiga, Japan; 2 Department of Aeronautical and Aviation Engineering, The Hong Kong Polytechnic University, 11 Yuk Choi Rd, Hung Hom, Kowloon, Hong Kong; 3 Department of Electronic Engineering, School of Engineering, Shibaura Institute of Technology, 3-7-5, Toyosu, Koto-ku, Tokyo 135-8548, Japan

**Keywords:** cyclist, orientation estimation, LiDAR, deep neural network

## Abstract

It is crucial for an autonomous vehicle to predict cyclist behavior before decision-making. When a cyclist is on real traffic roads, his or her body orientation indicates the current moving directions, and his or her head orientation indicates his or her intention for checking the road situation before making next movement. Therefore, estimating the orientation of cyclist’s body and head is an important factor of cyclist behavior prediction for autonomous driving. This research proposes to estimate cyclist orientation including both body and head orientation using deep neural network with the data from Light Detection and Ranging (LiDAR) sensor. In this research, two different methods are proposed for cyclist orientation estimation. The first method uses 2D images to represent the reflectivity, ambient and range information collected by LiDAR sensor. At the same time, the second method uses 3D point cloud data to represent the information collected from LiDAR sensor. The two proposed methods adopt a model ResNet50, which is a 50-layer convolutional neural network, for orientation classification. Hence, the performances of two methods are compared to achieve the most effective usage of LiDAR sensor data in cyclist orientation estimation. This research developed a cyclist dataset, which includes multiple cyclists with different body and head orientations. The experimental results showed that a model that uses 3D point cloud data has better performance for cyclist orientation estimation compared to the model that uses 2D images. Moreover, in the 3D point cloud data-based method, using reflectivity information has a more accurate estimation result than using ambient information.

## 1. Introduction

Understanding the behavior of bicycle riders is one of the essential factors of autonomous driving system. Non-motorized road users include pedestrians, cyclists, etc. [1]. However, while a lot of research has been conducted on pedestrian detection and behavior analysis [2,3], there is a lack of discussion on the estimation of a cyclist’s behavior. As cyclists have more dynamic behavior and higher speeds than pedestrians, cyclist safety is also a critical issue that needs to be discussed. According to various traffic-related reports, a significant number of traffic incidents involve cyclists. In 2021, 43.6% of traffic accidents involved bicycle riders in Tokyo, Japan. This percentage of cyclists involved in accidents is only increasing since 2016 [4]. Furthermore, the observatory in European Union countries showed that cyclist safety is not being improved in various countries. In EU27 countries, the number of fatalities in traffic crashes involving cyclists remained constant between 2010 and 2019, while the number of fatalities in crashes involving other road users has decreased [5]. As the autonomous driving system is being developed for transportation efficiency along with safer roads, estimating cyclist orientation could be a solution for improvement on cyclist safety.

In real traffic situations, various road users including automobiles, pedestrians and bicycles move toward different directions. The easiest and most direct communication method for cyclists is to use hand signals to indicate their moving intentions. Accordingly, there are related regulations in some countries to encourage cyclist’s hand signals [6,7]. However, such hand signals could be informative, but they are not efficient enough to be applied into real traffic interactions between bicycles and automobiles, as they require time for both cyclists and drivers to process the signals [8]. Thus, the autonomous driving system is expected to estimate cyclist’s behavior without signals sent intentionally by the cyclist. In this research, estimation on a cyclist’s body and head orientation is proposed to consider a cyclist’s natural behavior, instead of hand signals. The ongoing direction of a cyclist can be represented by his or her body on a bicycle, as a cyclist rides in a fixed posture on a bicycle. Moreover, a cyclist’s intention for changing direction can be represented by his or her head direction, which is the most predictable cue for a cyclist’s turn [9,10]. Thus, the head and body orientations are critical factors in cyclist behavior estimation, as they indicate the future trajectory.

Most of previous research on pedestrian and cyclist orientation estimation are conducted based on the RGB-camera sensor widely used in autonomous vehicles. Schulz et al. developed a system to localize head and estimate the head orientation [11,12]. The system has eight separate classifiers corresponding to eight different head orientations. The head localization and head orientation estimation were realized by comparing the output confidence values generated by all eight classifiers. Gandhi et al. used the famous Support Vector Machines (SVM) and Histograms of Oriented Gradient (HOG) to estimate the pedestrian body orientation [13]. Gu et al. proposed using human physical model constraint and temporal constraint to accurately estimate the joined body and head orientation of pedestrians and cyclists in video [14,15]. Flohr et al. presented a probabilistic framework for the joint estimation of pedestrian head and body orientation from a mobile stereo vision platform using the pictorial structure approach [16].

Recently, deep neural networks have been widely used for vision-based orientation estimation of pedestrians and cyclists. Raza et al. presented an appearance-based pedestrian head-pose and full-body orientation prediction by using grayscale image and employing a deep learning mechanism [17]. Abadi et al. proposed to estimate the cyclist head and body orientation using joined head map information generated from Openpose [18], and then used the joined head and body orientation to predict the crossing intention of cyclist [19,20]. In order to identify the cyclist heading and predict their intentions, Garcia et al. proposed a multi-class detection with eight classes according to orientations and presented a performance comparison for cyclist detection and orientation classification between the main deep-learning-based algorithms reported in the literature, such as SSD, Faster R-CNN and R-FCN [21].

However, there is a certain weakness in using a camera for pedestrian and cyclist orientation estimation. As a camera is hugely dependent on light variation, the estimation results from images taken by a camera can be unstable. Such a problem can be solved by using LiDAR (Light Detection and Ranging) sensor. Different from the passive sensor camera, LiDAR is an active sensor, and can emit pulsed light waves into surrounding objects and receive the bounced-back wave to calculate the distance. Hence, a 3D structure of the environment can be measured and presented as 3D point cloud [22] because the ranging mechanism of LiDAR sensor is independent from light sources in surrounding environments. Therefore, LiDAR sensor is not affected by light variation such as direct sunlight or night conditions where a camera struggles. Thus, pedestrian and cyclist detection has been conducted by using LiDAR sensor [23,24,25,26,27]. The camera can capture higher-resolution images of objects; on the other hand, LiDAR can acquire accurate 3D information of objects and has better performance in extreme light variation. Therefore, an integration of camera image and LiDAR information was proposed for pedestrian detection [28,29,30,31]. However, there are a few discussions for cyclist orientation estimation using LiDAR sensor. The autonomous vehicle needs to work in different light conditions. Accordingly, research on the use of a LiDAR-based perception system can support the current system that uses a camera only, or be a redundancy in cases where the camera-based perception system cannot work. As there has been research on camera-based cyclist orientation estimation, this research focuses on the methods of LiDAR-based cyclist orientation.

The contribution of this research is to propose two different methods for cyclist orientation estimation. The first method is to convert LiDAR data into three gray-scaled images for cyclist orientation classification. The second method is to use 3D point cloud data to represent LiDAR data for cyclist orientation estimation. Both of the proposed methods use a model ResNet50 deep neural network for orientation classification. Finally, the performances of the above two methods are compared in order to approach the most effective usage of LiDAR sensor data for cyclist orientation estimation. In the research, a cyclist dataset including multiple cyclists with different body and head orientation is developed. The experimental results proved that the 3D point cloud data-based method has a better performance for cyclist orientation estimation compared to the 2D image-based method. Moreover, in the 3D point cloud data-based method, using reflectivity information has a more accurate estimation result than using ambient information.

The rest of the paper is organized as follows: Section 2 describes the two proposed methods. Section 3 presents the experimental result, and the paper is concluded in Section 4.

## 2. Cyclist Orientation Estimation Based on 2D and 3D Methods

### 2.1. Definition for Cyclist Body and Head Orientations

**Figure 1:**
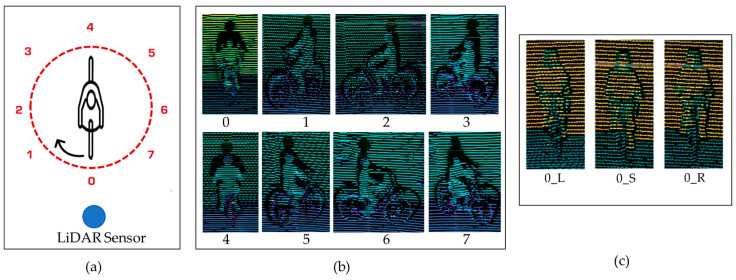
Demonstration of the definition of body orientation (**a**,**b**) and head orientation (**c**).

Following the conventional definition used in the research of camera-based orientation estimation [19,20], cyclist body orientation is labelled in eight classes and head orientation of body orientation is labelled in three classes in this research. The body orientation number is defined along clockwise, starting from an orientation facing the LiDAR sensor. The diagram for the eight body orientations of the cyclist in Bird’s Eye View (BEV) is shown on Figure 1a. In this diagram, the cyclist is in orientation “0”, and the arrow indicates the rotation direction for labeling. Figure 1b shows the example of 3D point cloud data for each body orientation taken by LiDAR sensor. Assuming that a LiDAR sensor has been equipped on a vehicle, the orientation “0” indicates that the cyclist is moving towards a vehicle and that the cyclist is moving closer to the vehicle. On the opposite direction, orientation “4” means that the cyclist is currently riding away from the vehicle. The orientations “2” and “6” indicate the situations of the cyclist riding perpendicular to the vehicle. The diagonal orientations “1” and orientation “7” indicates the situation where the cyclist is moving closer to the vehicle and there is a 45-degree angle between their moving directions. Similarly, the orientation “3” and orientation “5” refer to the behavior of the cyclist moving away from vehicle.

Detached from body orientation, the head orientation of a bicycle rider indicates the sight of the cyclist. Based on the head orientation, the future decisions of the cyclist are estimated. Considering the natural head rotation of a cyclist, the cyclist head orientation following the cyclist’s sight is labelled in three classes, which are L (Left), S (Straight) and R (Right). This head orientation is labelled in a cyclist’s perspective as well, meaning that the cyclist turns the head to the left, looks straight and turns the head to right. Thus, three head orientations are assigned to sub-classes for each of eight body orientations. This results in 24 classes that indicate the joined body and head orientation. Figure 1c demonstrates the head orientations in the event of “0” body orientation only, and the joined body and head orientations are labelled as “0_L”, “0_S” and “0_R” for the three postures of bicycle riders in body orientation “0” while looking on Left (L) side, looking Straight (S) and looking on Right (R) side.

This research works under the assumption that the cyclist has been detected from the LiDAR data. Some cyclist detection algorithms exist that can possibly fulfill this requirement [21,32]. In this research, the data of the cyclist’s area is manually cropped from the data collected by LiDAR sensor and used for orientation estimation.

### 2.2. 2D Image-Based Cyclist Orientation Estimation

**Figure 2:**
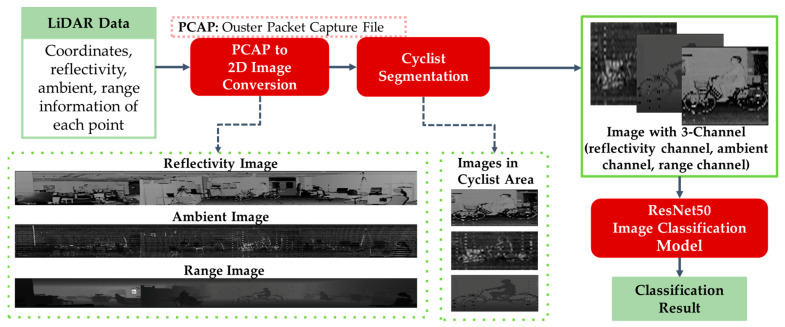
Flowchart of 2D image-based cyclist orientation estimation.

The proposed 2D image-based cyclist orientation estimation aims for classification of the cyclist head and body orientation based on the images generated from LiDAR sensor data. The methodology of this system is to transform information taken by LiDAR sensor into image format and classify cyclist images into different joined body and head orientations. The flowchart of the image-based orientation estimation method is shown in Figure 2, in which the red boxes represent the main stages of the method. Moreover, the solid green boxes represent the input and output of each stage. Additionally, the images in the dotted green boxes illustrate the examples of generated images in each main stage.

The first stage of the proposed method is a data preprocessing step that converts a raw datum captured from the LiDAR sensor to image. The data format of the LiDAR sensor varies depending on the LiDAR sensor manufacturer, and the LiDAR sensor used in this research records a sequence of sensor packets to a PCAP (Packet Capture) file [33]. Each sensor packet corresponds to one scan for the surrounding environment. The point cloud data in each scan contains the coordinates (x, y, z) of each point with four data layers: range, signal, reflectivity and ambient. Each data layer contains information that is not able to be captured from an RGB camera, which is a key information for data analysis in 3D space [34].

The range information of the point represents the distance from the sensor by calculating the travel time of the laser light wave. The signal information represents the strength of the light returned to the sensor for a given point. The reported reflectivity is a byproduct of range and signal that gives the user an indication of the target reflectivity. The ambient information denotes the strength of sunlight collected for a given point, also expressed in the quantity of photons detected that were not produced by the sensor’s laser pulse. The signal information varies with range (objects farther away return less light), and ambient data varies with sunlight levels. On the other hand, the reflectivity data are consistent across lighting conditions and range. Therefore, reflectivity is the only piece of data that contains information about the properties of the object, which is not light [34]. To avoid information redundancy, the signal information is not employed in the system, since the signal varies with range information. Therefore, the coordinates, range, reflectivity and ambient data of each point on the point cloud are utilized for the proposed image-based cyclist orientation estimation system.

For the image conversion, the 3D data are projected into 2D. The 3D point cloud captured from LiDAR sensor is perfectly 1:1 spatial correspond [35]. Hence, the images can be generated by analyzing the coordinates of each point and assign values to each pixel. This implies that each point on the point cloud is projected as each pixel on the images. Since the LiDAR sensor used in this research has a vertical resolution of 64 and a resolution of 2048 for 360-degree, there are 131,072 points captured on each scan, which is the same as the length of returned lists of data layers. These returned lists are combined into a 3D array with a size of (2048 × 64 × 6). This array represents a single frame of the captured sequence, and each element contains values of x, y, z, range, ambient and reflectivity. Hence, this array is divided into three arrays that each represents an image of a frame. As the spatial correspondence of the points is perfect, it can be directly projected onto a 2D array with a size of (2048 × 64) based on the position on the 3D array. Each converted pixel of a 1D array contains range, ambient and reflectivity values for arrays of range image, ambient image and reflectivity image, respectively. Since the converted image arrays contain only one value per pixel, the generated images are gray-scale images with one channel. Figure 2 shows the examples of generated range, ambient and reflectivity images.

**Figure 3:**
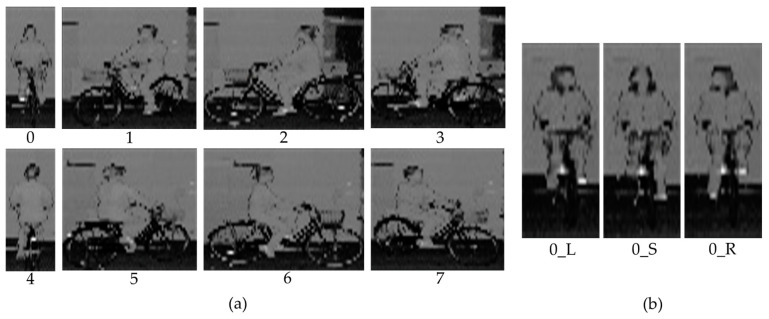
Reflectivity images of cyclist with different body orientations (**a**) and head orientations (**b**). (In addition to the reflectivity information, ambient and range are also used in 2D image-based cyclist orientation estimation).

The second stage of the proposed method is to segment the cyclist area from the range, ambient and reflectivity images. This research focuses on the cyclist orientation estimation, and assumes that cyclists have been detected by other methods, for example, applying YOLO [36] on generated gray scale images [35]. Thus, the cyclist area is manually cropped in this research. Figure 3a shows the example reflectivity images of cyclists with different body orientations, and Figure 3b demonstrates the reflectivity images for three head orientations in the case of “0” body orientation.

The ultimate goal of the 2D image-based cyclist orientation estimation system is to predict and classify the body and head orientation of cyclists based on images generated from LiDAR sensor data. In this research, a Residual Neural Network (ResNet) model with 50 convolution layers is used for this goal. Compared to traditional CNNs, ResNet can overcome the “vanishing gradient” problem. Therefore, it can construct networks with thousands of convolutional layers, and outperform shallower networks. Since ResNet has deep architecture and good performance for image recognition, it is widely used in the task of image classification. The input images of the image classification model are the range image, ambient image and reflectivity image of a cyclist from LiDAR sensor data. The desired input size of the model is (224 × 224) [37]. Therefore, the images are resized to be in the same size of (224 × 224). In addition, the three images are concatenated to be in one data array, which has array size of (224 × 224 × 3), and sent to the ResNet50 model to estimate the joined body and head orientation of cyclist. The experimental result of the 2D image-based method will be represented in Section 3.

### 2.3. 3D Point Cloud-Based Cyclist Orientation Estimation

**Figure 4:**
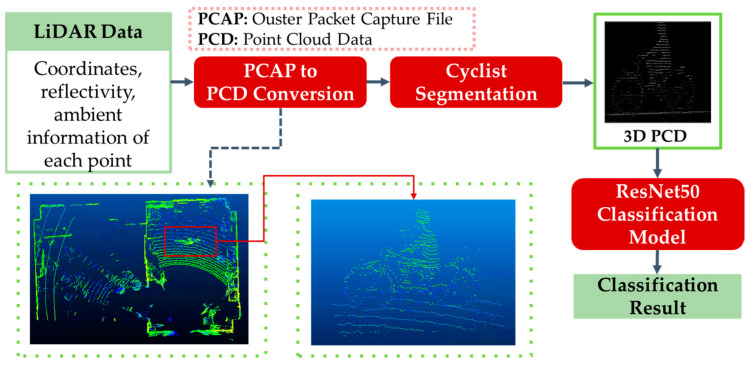
Flowchart of 3D point-cloud-based cyclist orientation estimation.

The basic methodology of the 3D point-cloud-based cyclist orientation estimation is similar to the 2D image-based method. However, this point-cloud-based method represents the information of a cyclist using 3D point cloud data instead of a 2D image. The flowchart of the proposed 3D point-cloud-based cyclist orientation estimation is illustrated in Figure 4. In Figure 4, the important stages are indicated in a red box. Moreover, the input and output data of each stage are indicated in green color.

In order to process the point cloud data in each scan, it is necessary to convert it from a packet file of the LiDAR sensor, PCAP, to a Point Cloud Data format, PCD. The point clouds stored in PCD file contains a collection of 3D coordinates (x, y, z) with other data layers. In this proposed system, the data layers of reflectivity information and ambient information are chosen to be utilized, while excluding range information. This is because the range information represents the 3D shape of an object using the distance of the points from the sensor to the object, with the 3D shape of the object also able be represented by coordinates (x, y, z). Therefore, using a range data layer is a redundancy in the information. Thus, reflectivity and ambient information are used in the proposed system. Hence, there are two types of input to the system, which are two arrays containing 3D coordinates with reflectivity and ambient each, respectively. After each array is used as an input to the orientation classification model, the results of each case can be compared to understand the best data layer for this 3D point-cloud-based method. Finally, the arrays are saved as a PCD file by the point cloud library [38].

After the PCD conversion stage, cyclist segmentation is performed to acquire point cloud data in the cyclist area. This research also assumes that cyclists have been detected by other methods. For example, it is possible to convert a 3D point cloud into RGB-map in Bird’s Eye View (BEV) and implement image object detection algorithm, YOLO, on the BEV image [21]. Following this idea, the point cloud data of the cyclist is segmented from BEV in the software CloudCompare. Figure 4 shows the BEV of point cloud data and the front view of the segmented cyclist area.

After segmentation process, the point cloud data are no longer in a vertical size of 64 and a horizontal size of 2048. Instead, the size of the point cloud data is dependent on the posture of the cyclist, especially the body orientation. On the other words, the number of points on the point cloud is also inconsistent across the body orientation. As the point cloud data is an input to the classification model, the size of the data should be equal. Therefore, the point cloud data are normalized in a vertical size of 224, a horizontal size of 224 and depth of 50, followed by the input configuration of classification model.

The segmented and normalized point cloud data of a cyclist is returned as an output from the previous data preprocessing stages. Hence, they are input to the point cloud data classification model for cyclist body and head orientation estimation. The purpose of this research is to compare the cyclist orientation estimation results between converting LiDAR data to a 2D image and using the 3D point cloud data from the sensor. Hence, the same classification model for the image-based method is used in the point-cloud-based method, which is ResNet50. The point cloud data in 3D array is input to ResNet50 in a size of (224 × 224 × 50). Since elements of points on the point cloud data indicate their reflectivity or ambient value, the values of points are processed as a value of a pixel in the case of images. For a clear comparison between the two different usages of LiDAR sensor data, the same architecture is used with a slight modification on input configuration for the 3D point-cloud-based method. The experimental result of the 3D point-cloud-based method will be represented in Section 3.

## 3. Experiments

### 3.1. Data Collection

In this research, the LiDAR sensor data of a cyclist with different body and head orientations is required. The LiDAR sensor that is used for the experiment is Ouster OS1-64. The LiDAR sensor used in the experiment has 64 channels of resolution, which indicates the number of beams that are sent at once. Moreover, it has a horizontal resolution of 2048, which means that the 64 channel beams are sent 2048 times per frame. Thus, it records 131,072 points per frame, and it can capture 10 frames per second. The data are collected indoor with a bicycle in a fixed position. The LiDAR sensor is installed about 7 m away from the bicycle’s position. Hence, the bicycle is rotated clockwise within the target body orientation. In the data collection, a total of 12 students participated as cyclists. Participants are asked to ride a bicycle by pedaling on a bicycle in a fixed position.

Twelve participants are asked to do the pedaling, and they are asked to turn their head three times per body orientation. To capture a clear posture, the cyclists are supposed to turn their head completely to each direction and maintain a solid head orientation while the data are taken. The cyclists’ data are taken for 5 s per head and body orientation, which indicates 50 frames per joined body and head orientation. Finally, there are about 14,000 frames taken for joined body and head orientation in total. Table 1 is shown below to represent the number of frames taken for each joined body and head orientation with the total number of each body orientation.

The datasets of cyclists are divided into training datasets and validation datasets for cross validation. The 4-fold cross validation is used in evaluation. The training process has an epoch of 40, and the validation process compares the predicted labels of a cyclist’s orientation with the ground truth labels for evaluation. For the experiments, a computer with an Intel Core i9-9900K CPU running at 3.60 GHz, an NVIDIA GeForce RTX 2070-Super GPU, and a RAM memory of 16 GB is adopted. 

### 3.2. Experimental Results

This research presents three classification results which are the results of the 2D image-based method, the 3D point cloud with reflectivity-value-based method and 3D point cloud with ambient-value-based method. The main evaluation method of the classification model is prediction accuracy, as it is a direct and quantitative measurement of the classification. However, such an evaluation method by comparing accuracy can lead to unclear classification errors between the classes. Therefore, a confusion matrix is additionally used to present the performance of the classification. The confusion matrix is a table that records the correctness of predicted labels with true labels in percentage. Since there are multiple numbers of orientation classes, the multi-class confusion matrix is used as a performance indicator. 

**Figure 5:**
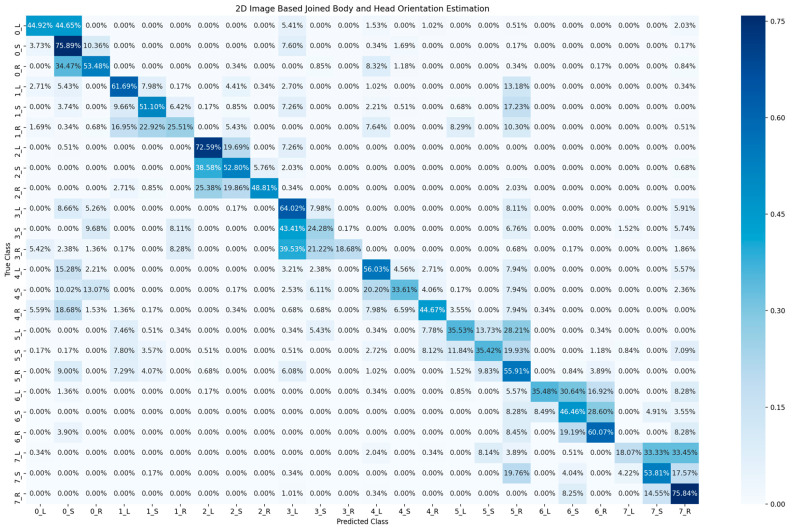
Confusion matrix of cyclist joined body and head orientation estimation using 2D images.

**Figure 6:**
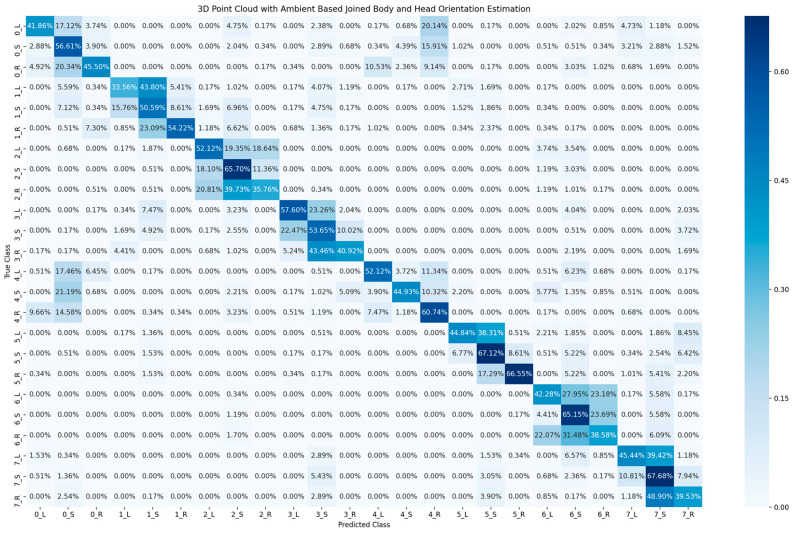
Confusion matrix of cyclist joined body and head orientation estimation using 3D point cloud data with ambient.

**Figure 7:**
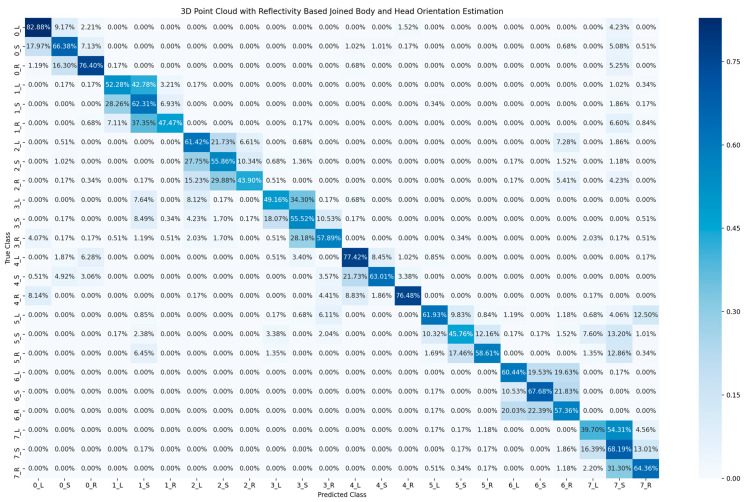
Confusion matrix of cyclist joined body and head orientation estimation using 3D point cloud data with reflectivity.

Table 2 shows the accuracy of classification for the cyclist joined body and head orientation estimation of the two proposed methods. The confusion matrix in Figure 5 illustrates the classification results of the 2D-based method for joined body and head orientation estimation. In Figure 6 and Figure 7, the classification results of the 3D-based method with ambient and reflectivity as input are shown, respectively. Obviously, the classification accuracy of joined body and head orientation is higher in the 3D point-cloud-based method in general. Moreover, using reflectivity resulted in better accuracy than using ambient.

**Figure 8:**
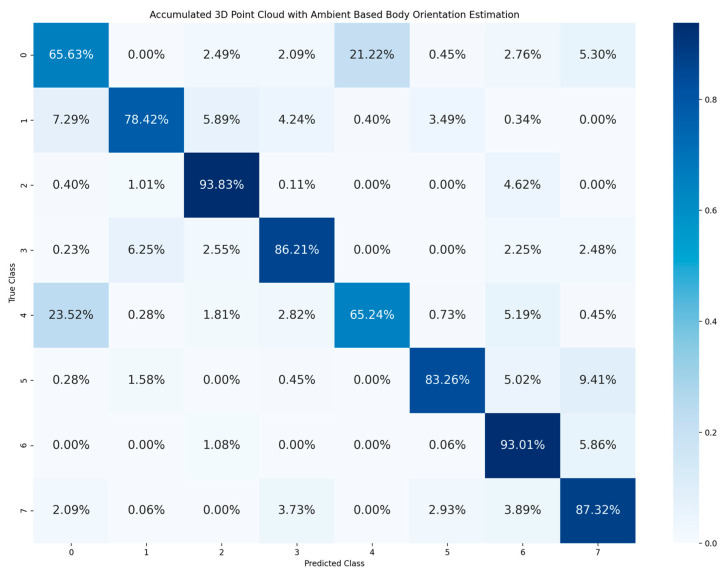
Confusion matrix of body orientation estimation accumulated from joined body and head orientation estimation using 3D point cloud data with ambient.

**Figure 9:**
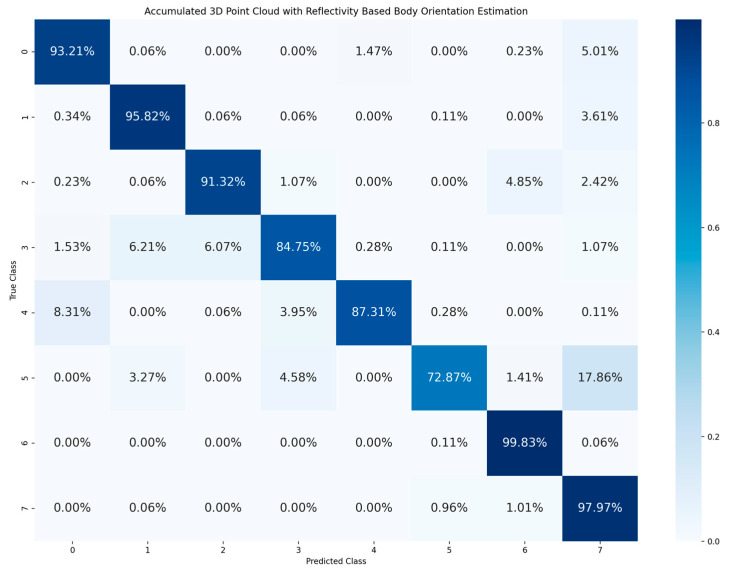
Confusion matrix of body orientation estimation accumulated from joined body and head orientation estimation using 3D point cloud data with reflectivity.

Furthermore, the results of the 3D-based method are accumulated from 24 joined body and head orientation classes into 8 body orientation classes in Figure 8 and Figure 9. Once the estimation result of joined body and head orientation estimation is summarized into a body orientation estimation result, it is obvious that there are much higher correction rates and stability than in joined body and head orientation estimation. Since the joined body and head orientation estimation aims for more detailed behavior of cyclists, the head orientation is added as a sub-class to the body orientation. Hence, there are more classes for classification, numbering 24 in total. This increase in number of classes for more detailed posture analysis resulted in a decrease in estimation accuracy. Moreover, the head orientation is not well estimated since the accuracy dropped when head orientation is added as a sub-class to the body orientation. More specifically, for the estimation accuracy calculated after the accumulation for eight body orientation, the 3D point cloud data with ambient have 81.62% accuracy and 3D point cloud data with reflectivity have 90.34% accuracy. In this research, all experiments were performed in an indoor environment with artificial illumination, which is better than the illumination condition during night in an outdoor environment. However, using ambient information cannot have a better performance than using reflectivity information for body orientation estimation. 

**Figure 10:**
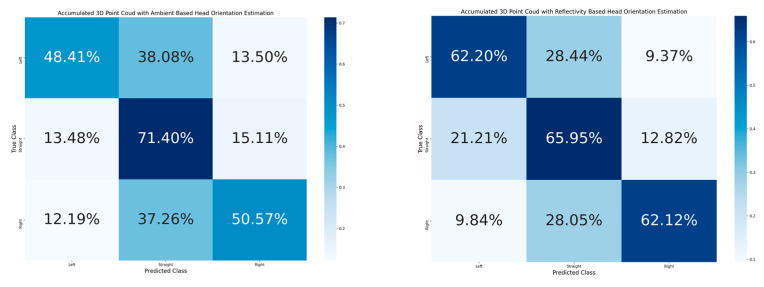
**Left**: Confusion matrix of head orientation estimation accumulated from joined body and head orientation estimation using 3D point cloud data with ambient. **Right**: Confusion matrix of head orientation estimation accumulated from joined body and head orientation estimation using 3D point cloud data with reflectivity.

In order to have analysis on head orientation specifically, the results of the 3D-based method are accumulated from 24 joined body and head orientation classes into 3 head orientation classes in Figure 10. The confusion matrix of three head classes shows more unstable estimation results than when they are accumulated into eight body orientation classes. The head orientation estimation correction rate of 3D point cloud data with ambient shows 56.79% accuracy, while 3D point cloud data with reflectivity shows a correction rate of 63.42%. The estimation resulted in better accuracy when the head orientation is eliminated from the joined body and head orientation estimation. Therefore, there is a challenge for head orientation estimation with both the proposed 2D image-based method and the 3D point-cloud-based method. 

### 3.3. Discussions

**Figure 11:**
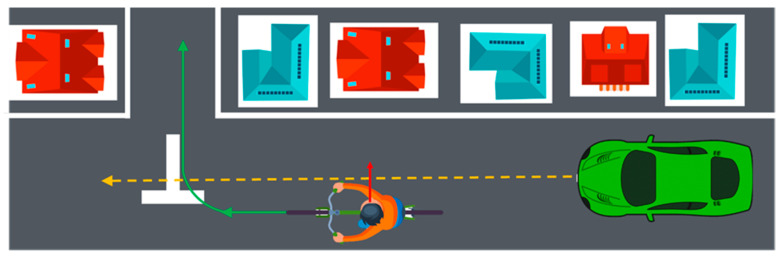
Demonstration of a scenario where a cyclist is turning his or her head before crossing the road.

In this research, the cyclist head orientation is labeled into three classes: L (Left), S (Straight) and R (Right). The three classes correspond to the three cases: cyclist turns head to the left, looks straight and turns head to the right. In fact, head movement is the most reliable indicator for detecting cyclist intention when they are about to make a turn. Figure 11 demonstrates one of the most dangerous scenarios in the real traffic situation. The cyclist is planning to turn right and cross the road, as indicated by the green arrow trajectory line. Usually, cyclists maximally turn their heads (about 90 degrees, as indicated by the red arrow) to check the situation behind them before they turn their bicycle. To simulate this situation, head orientations L and R are defined as a 90-degree difference from the body orientation. The head orientation S means that the body and head orientations are the same (0-degree difference). In fact, there are more complicated cases in real traffic situations. This research uses three cases to test the feasibility of head orientation estimation.

The experimental result indicates that the accuracy of the head orientation is relatively low and needs to be improved. Recently, the deep-learning-based super-resolution technique is not only used for image processing, but also extended for point cloud processing. It is possible to employ the super-resolution [39,40] techniques to increase the resolution of a cyclist to improve the accuracy of the head orientation estimation.

As we can see from Figure 8 and Figure 9, misclassification often happens between body orientation 0 and body orientation 4. This is because the LiDAR data of these two classes are similar. However, when the body orientation estimation is performed based on an RGB camera in the ideal light condition, the misclassifications between body orientation 0 and orientation 4 are few [20] because the cyclist’s face can be clearly represented in an RGB image and used as the main feature to distinguish the two classes. However, the resolution of LiDAR sensor is low and cannot represent the face clearly. In fact, distinguishing these two classes is significant for cyclist safety; one possible solution is to use super-resolution techniques [39,40] to increase the resolution of LiDAR data. Another way is to track the trajectory of the cyclist in order to understand whether the cyclist is moving towards the vehicle or riding away from the vehicle.

In the experiment, the distance between cyclists and LiDAR sensor is around 7 m. Theoretically, when cyclists go far from the sensor, the classification capability of the developed system should decrease. This problem can be solved by increasing the resolution of LiDAR sensor data, e.g., using super-resolution [39,40] techniques.

The paper proposes two different methods for cyclist orientation estimation, aiming to illustrate the comparison between the two proposed methods. The dataset used in this research only contains cyclists without carrying bags. However, cyclists sometimes may carry bags or other objects in real traffic situations. When the proposed method is used in real applications, the dataset for training should be enriched to include different types of cyclists, e.g., those carrying backpacks or sling bags. By adding the different types of sample data, the retrained model is expected to recognize the orientation of cyclists with carrying bags.

## 4. Conclusions

In this research, a system for cyclist body and head orientation estimation using LiDAR sensor data is proposed. The ultimate goal of the proposed system is to approach the most effective usage of LiDAR sensor data for cyclist body orientation estimation. The first method suggested an approach to convert LiDAR sensor data into three different gray-scaled images by utilizing the data layers of range, ambient and reflectivity. Hence, the method used the images together as a three-channel image as an input to orientation classification model. On the other hand, the second method proposed using the LiDAR sensor as a 3D point cloud data with each point containing an ambient layer or a reflectivity layer for the orientation classification. The evaluation of the proposed system is based on the classification accuracy and confusion matrix. The results of the experimentation proved that the proposed 3D point-cloud-based cyclist orientation system leads to better prediction results for the cyclist joined body and head orientation estimation than the 2D image-based method. The 2D image-based method resulted in 47% accuracy, the 3D point cloud data with ambient in 51% accuracy and the 3D point cloud data with reflectivity in 60% accuracy. Therefore, the best usage of LiDAR data for cyclist orientation estimation is to utilize the data into 3D point cloud data with a reflectivity layer of each point. Moreover, there is a challenge regarding joined body and head orientation estimation which resulted in much less prediction accuracy than body orientation alone.

In the future, the super-resolution technique will be adopted to improve the accuracy of orientation estimation, especially for the head orientation estimation.

## Figures and Tables

**Table 1:** Number of frames in dataset for joined body and head orientation.

Head Orientation	Body Orientation								Total
	0	1	2	3	4	5	6	7	
**Left**	590	591	591	592	589	591	589	592	
**Straight**	589	589	589	589	592	590	594	591	
**Right**	589	592	590	589	591	592	591	592	
**Sub-total**	1768	1772	1770	1770	1772	1773	1774	1775	14,174

**Table 2:** Cyclist Joined Body and Head Orientation Estimation Accuracy of Different Usage of LiDAR data.

	2D Image Based Method	3D Point Cloud Based Methods	
		Ambient	Reflectivity
**Accuracy**	47.69%	50.96%	60.52%

## Data Availability

The data are not publicly available due to restrictions on privacy.
